# 3-(Piperidin-1-yl)-6-(1*H*-pyrazol-1-yl)pyridazine

**DOI:** 10.1107/S1600536810016491

**Published:** 2010-05-08

**Authors:** Abdul Qayyum Ather, Onur Şahin, Islam Ullah Khan, Misbahul Ain Khan, Orhan Büyükgüngör

**Affiliations:** a Department of Chemistry, Islamia University, Ba-hawalpur, Pakistan and Applied Chemistry Research Center, PCSIR Laboratories Complex, Lahore 54600, Pakistan; b Department of Physics, Ondokuz Mayıs University, TR-55139 Samsun, Turkey; c Materials Chemistry Laboratory, Department of Chemistry, GC University, Lahore 54000, Pakistan; d Institute of Chemistry, University of the Punjab, Lahore 54000, Pakistan

## Abstract

In the title compound, C_12_H_15_N_5_, the piperidine ring adopts a chair conformation with the substituent C atom in an equatorial site and the dihedral angle between the pyridazine and pyrazole ring planes is 10.36 (2)°.

## Related literature

For related structures, see: Blake *et al.* (2002 ▶); Ather *et al.* (2009 ▶).
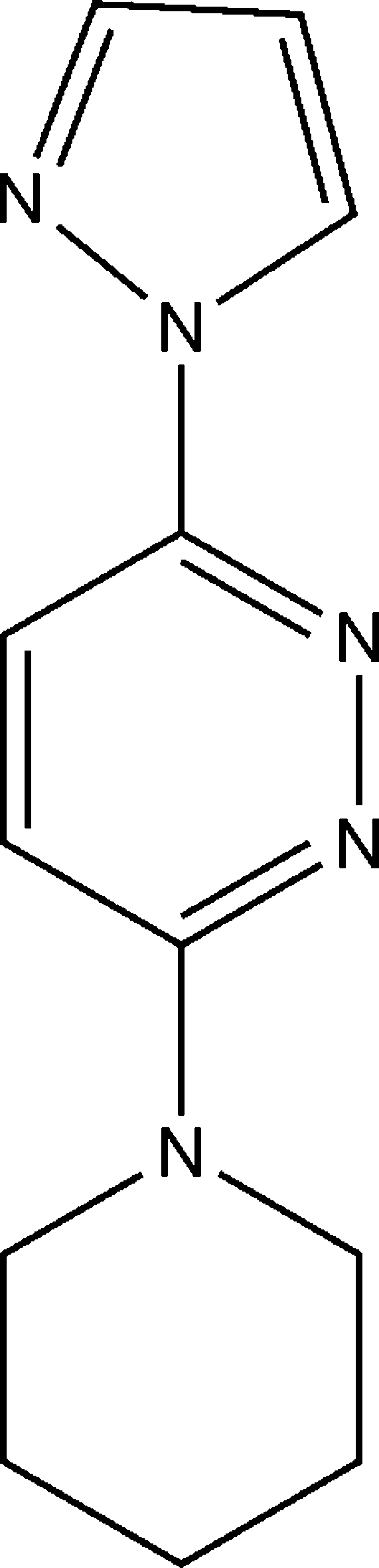


## Experimental

### 

#### Crystal data

C_12_H_15_N_5_
*M* *_r_* = 229.29
Monoclinic, 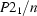

*a* = 5.9665 (6) Å
*b* = 20.189 (3) Å
*c* = 9.9695 (13) Å
β = 103.230 (7)°
*V* = 1169.0 (2) Å^3^
*Z* = 4
Mo *K*α radiation
μ = 0.08 mm^−1^
*T* = 296 K
0.31 × 0.25 × 0.22 mm

#### Data collection

Bruker APEXII CCD diffractometer
11710 measured reflections
2674 independent reflections
1282 reflections with *I* > 2σ(*I*)
*R*_int_ = 0.054

#### Refinement

*R*[*F* ^2^ > 2σ(*F* ^2^)] = 0.058
*wR*(*F* ^2^) = 0.182
*S* = 1.01
2674 reflections
154 parameters
H-atom parameters constrained
Δρ_max_ = 0.18 e Å^−3^
Δρ_min_ = −0.21 e Å^−3^

### 

Data collection: *APEX2* (Bruker, 2007 ▶); cell refinement: *SAINT* (Bruker, 2007 ▶); data reduction: *SAINT*; program(s) used to solve structure: *SHELXS97* (Sheldrick, 2008 ▶); program(s) used to refine structure: *SHELXL97* (Sheldrick, 2008 ▶); molecular graphics: *ORTEP-3* (Farrugia, 1997 ▶); software used to prepare material for publication: *WinGX* (Farrugia, 1999 ▶).

## Supplementary Material

Crystal structure: contains datablocks global, I. DOI: 10.1107/S1600536810016491/hb5436sup1.cif

Structure factors: contains datablocks I. DOI: 10.1107/S1600536810016491/hb5436Isup2.hkl

Additional supplementary materials:  crystallographic information; 3D view; checkCIF report

## Figures and Tables

**Fig. 1.**
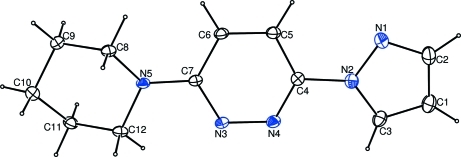
A view of (I), showing 30% displacement ellipsoids.

## supplementary crystallographic information

### Comment

As part of our onging studies of azolylpyridazines (Ather *et al.*, 2009), we now report the synthesis and structure of the title compound, (I).

Compound (I) consists of a pyridazine ring with piperidine and pyrazole substituents at the 3- and 6-positions, respectively (Fig. 1). Least-squares mean-plane calculations for the pyridazine (N3/N4/C4/C5/C6/C7) and pyrazole (N1/N2/C3/C1/C2) rings show that these are approximately planar, with respective maximum deviations of 0.0042 (16)Å for atom C7 and 0.0026 (19)Å for atom C2. The dihedral angle between the pyridazine and pyrazole ring planes is 10.36 (2)°. The piperidine ring in (I) adopts a chair conformation. The N5—C7 and N2—C4 bond lengths indicate significant single-bond character, whereas the N3═C7 and N4═C4 bond lengths are indicative of significant double-bond character. The N1—N2 and N3—N4 bond lengths [1.357 (3)Å and 1.353 (3) Å, respectively] agree with the corresponding distances in 3,4,6-Tris(pyrazol-1-yl)pyridazine (Blake *et al.*, 2002).

### Experimental

A mixture of 1.0 g (0.18 mmol) of 3-chloro-6-(1 H-Pyrozol-1-yl) pyridazine and 5 ml of piperidine was refluxed for 2 h, concentrated under vacuum, cooled and added to cooled water. The ppt filtered dried and recrystallized from benzene to give colourless prisms of (I) (m.p. 383-384 K).

### Refinement

All H atoms attached to C atoms were refined using a riding model [C—H = 0.93Å and U_iso_(H) = 1.2U_eq_(C) for aromatic H atoms and C—H = 0.97Å and U_iso_(H) = 1.2U_eq_(C) for metyhlene H atoms].

### Crystal data

**Table:**

C_12_H_15_N_5_	*F*(000) = 488
*M**_r_* = 229.29	*D*_x_ = 1.303 Mg m^−^^3^
Monoclinic, *P*2_1_/*n*	Mo *K*α radiation, λ = 0.71073 Å
Hall symbol: -P 2yn	Cell parameters from 1639 reflections
*a* = 5.9665 (6) Å	θ = 2.3–21.3°
*b* = 20.189 (3) Å	µ = 0.08 mm^−^^1^
*c* = 9.9695 (13) Å	*T* = 296 K
β = 103.230 (7)°	Prism, colourless
*V* = 1169.0 (2) Å^3^	0.31 × 0.25 × 0.22 mm
*Z* = 4	

### Data collection

**Table:**

Bruker APEXII CCD diffractometer	1282 reflections with *I* > 2σ(*I*)
Radiation source: fine-focus sealed tube	*R*_int_ = 0.054
graphite	θ_max_ = 27.5°, θ_min_ = 2.3°
phi and ω scans	*h* = −7→7
11710 measured reflections	*k* = −26→26
2674 independent reflections	*l* = −12→12

### Refinement

**Table:**

Refinement on *F*^2^	Primary atom site location: structure-invariant direct methods
Least-squares matrix: full	Secondary atom site location: difference Fourier map
*R*[*F*^2^ > 2σ(*F*^2^)] = 0.058	Hydrogen site location: inferred from neighbouring sites
*wR*(*F*^2^) = 0.182	H-atom parameters constrained
*S* = 1.01	*w* = 1/[σ^2^(*F*_o_^2^) + (0.0863*P*)^2^] where *P* = (*F*_o_^2^ + 2*F*_c_^2^)/3
2674 reflections	(Δ/σ)_max_ < 0.001
154 parameters	Δρ_max_ = 0.18 e Å^−^^3^
0 restraints	Δρ_min_ = −0.21 e Å^−^^3^

### Special details

**Table:**

Geometry. All esds (except the esd in the dihedral angle between two l.s. planes) are estimated using the full covariance matrix. The cell esds are taken into account individually in the estimation of esds in distances, angles and torsion angles; correlations between esds in cell parameters are only used when they are defined by crystal symmetry. An approximate (isotropic) treatment of cell esds is used for estimating esds involving l.s. planes.
Refinement. Refinement of *F*^2^ against ALL reflections. The weighted *R*-factor wR and goodness of fit *S* are based on *F*^2^, conventional *R*-factors *R* are based on *F*, with *F* set to zero for negative *F*^2^. The threshold expression of *F*^2^ > σ(*F*^2^) is used only for calculating *R*-factors(gt) etc. and is not relevant to the choice of reflections for refinement. *R*-factors based on *F*^2^ are statistically about twice as large as those based on *F*, and *R*- factors based on ALL data will be even larger.

### Fractional atomic coordinates and isotropic or equivalent isotropic displacement parameters (Å^2^)

**Table:**

	*x*	*y*	*z*	*U*_iso_*/*U*_eq_	
C1	0.3406 (6)	0.72572 (15)	0.3173 (3)	0.0738 (9)	
H1	0.4014	0.6901	0.2782	0.089*	
C2	0.1112 (6)	0.74333 (16)	0.2984 (3)	0.0756 (9)	
H2	−0.0087	0.7203	0.2415	0.091*	
C3	0.4568 (5)	0.77160 (14)	0.4052 (3)	0.0639 (8)	
H3	0.6155	0.7740	0.4384	0.077*	
C4	0.3331 (4)	0.86969 (12)	0.5224 (2)	0.0452 (6)	
C5	0.1481 (4)	0.90081 (13)	0.5592 (3)	0.0501 (7)	
H5	−0.0019	0.8857	0.5274	0.060*	
C6	0.1955 (4)	0.95375 (14)	0.6429 (2)	0.0496 (7)	
H6	0.0779	0.9767	0.6699	0.060*	
C7	0.4275 (4)	0.97398 (12)	0.6893 (2)	0.0415 (6)	
C8	0.3360 (4)	1.08147 (13)	0.7801 (3)	0.0540 (7)	
H8A	0.1774	1.0665	0.7559	0.065*	
H8B	0.3581	1.1125	0.7098	0.065*	
C9	0.3800 (4)	1.11603 (15)	0.9163 (3)	0.0638 (8)	
H9A	0.3378	1.0870	0.9840	0.077*	
H9B	0.2842	1.1553	0.9085	0.077*	
C10	0.6306 (4)	1.13584 (14)	0.9652 (3)	0.0674 (8)	
H10A	0.6698	1.1689	0.9037	0.081*	
H10B	0.6567	1.1548	1.0569	0.081*	
C11	0.7792 (4)	1.07512 (14)	0.9672 (3)	0.0580 (8)	
H11A	0.9401	1.0879	0.9932	0.070*	
H11B	0.7501	1.0443	1.0359	0.070*	
C12	0.7328 (4)	1.04124 (14)	0.8292 (3)	0.0530 (7)	
H12A	0.7779	1.0702	0.7623	0.064*	
H12B	0.8244	1.0012	0.8360	0.064*	
N1	0.0810 (4)	0.79630 (13)	0.3697 (2)	0.0692 (7)	
N2	0.2982 (4)	0.81341 (11)	0.4357 (2)	0.0536 (6)	
N3	0.5964 (3)	0.94188 (11)	0.6493 (2)	0.0493 (6)	
N4	0.5475 (3)	0.88877 (11)	0.5649 (2)	0.0523 (6)	
N5	0.4896 (3)	1.02450 (10)	0.7825 (2)	0.0449 (5)	

### Atomic displacement parameters (Å^2^)

**Table:**

	*U*^11^	*U*^22^	*U*^33^	*U*^12^	*U*^13^	*U*^23^
C1	0.099 (3)	0.052 (2)	0.072 (2)	0.0073 (17)	0.0227 (18)	−0.0060 (17)
C2	0.093 (3)	0.063 (2)	0.070 (2)	−0.0127 (18)	0.0162 (17)	−0.0139 (18)
C3	0.0711 (19)	0.0549 (19)	0.0662 (19)	0.0141 (15)	0.0166 (15)	−0.0039 (16)
C4	0.0482 (14)	0.0451 (16)	0.0416 (14)	0.0045 (11)	0.0088 (11)	0.0059 (12)
C5	0.0386 (13)	0.0639 (19)	0.0480 (15)	0.0021 (12)	0.0104 (11)	0.0014 (14)
C6	0.0327 (12)	0.0650 (19)	0.0517 (16)	0.0071 (12)	0.0108 (10)	−0.0019 (14)
C7	0.0343 (12)	0.0508 (16)	0.0404 (14)	0.0054 (11)	0.0106 (9)	0.0055 (12)
C8	0.0348 (13)	0.0593 (18)	0.0672 (18)	0.0064 (12)	0.0104 (11)	−0.0022 (15)
C9	0.0484 (16)	0.0595 (19)	0.086 (2)	0.0036 (13)	0.0211 (13)	−0.0177 (16)
C10	0.0549 (17)	0.064 (2)	0.084 (2)	−0.0063 (14)	0.0176 (14)	−0.0157 (16)
C11	0.0395 (13)	0.071 (2)	0.0610 (18)	−0.0092 (13)	0.0070 (11)	−0.0054 (15)
C12	0.0322 (12)	0.0668 (19)	0.0600 (17)	0.0016 (12)	0.0107 (10)	0.0036 (14)
N1	0.0619 (15)	0.0732 (18)	0.0691 (17)	−0.0082 (12)	0.0077 (12)	−0.0157 (14)
N2	0.0590 (13)	0.0462 (14)	0.0545 (14)	0.0035 (11)	0.0110 (10)	0.0021 (11)
N3	0.0378 (11)	0.0543 (14)	0.0583 (13)	0.0065 (9)	0.0161 (9)	−0.0031 (11)
N4	0.0433 (12)	0.0542 (14)	0.0610 (14)	0.0082 (10)	0.0156 (10)	−0.0008 (12)
N5	0.0286 (9)	0.0566 (14)	0.0496 (12)	0.0048 (9)	0.0090 (8)	−0.0008 (11)

### Geometric parameters (Å, °)

**Table:**

C1—C3	1.352 (4)	C8—C9	1.496 (4)
C1—C2	1.384 (4)	C8—H8A	0.9700
C1—H1	0.9300	C8—H8B	0.9700
C2—N1	1.319 (4)	C9—C10	1.516 (3)
C2—H2	0.9300	C9—H9A	0.9700
C3—N2	1.353 (3)	C9—H9B	0.9700
C3—H3	0.9300	C10—C11	1.510 (4)
C4—N4	1.310 (3)	C10—H10A	0.9700
C4—C5	1.391 (3)	C10—H10B	0.9700
C4—N2	1.414 (3)	C11—C12	1.504 (3)
C5—C6	1.346 (3)	C11—H11A	0.9700
C5—H5	0.9300	C11—H11B	0.9700
C6—C7	1.415 (3)	C12—N5	1.458 (3)
C6—H6	0.9300	C12—H12A	0.9700
C7—N3	1.334 (3)	C12—H12B	0.9700
C7—N5	1.372 (3)	N1—N2	1.357 (3)
C8—N5	1.467 (3)	N3—N4	1.353 (3)
			
C3—C1—C2	104.9 (3)	C8—C9—H9B	109.3
C3—C1—H1	127.6	C10—C9—H9B	109.3
C2—C1—H1	127.6	H9A—C9—H9B	107.9
N1—C2—C1	112.8 (3)	C11—C10—C9	108.8 (2)
N1—C2—H2	123.6	C11—C10—H10A	109.9
C1—C2—H2	123.6	C9—C10—H10A	109.9
C1—C3—N2	106.9 (3)	C11—C10—H10B	109.9
C1—C3—H3	126.5	C9—C10—H10B	109.9
N2—C3—H3	126.5	H10A—C10—H10B	108.3
N4—C4—C5	123.9 (2)	C12—C11—C10	111.9 (2)
N4—C4—N2	115.5 (2)	C12—C11—H11A	109.2
C5—C4—N2	120.7 (2)	C10—C11—H11A	109.2
C6—C5—C4	117.1 (2)	C12—C11—H11B	109.2
C6—C5—H5	121.5	C10—C11—H11B	109.2
C4—C5—H5	121.5	H11A—C11—H11B	107.9
C5—C6—C7	118.9 (2)	N5—C12—C11	111.04 (19)
C5—C6—H6	120.6	N5—C12—H12A	109.4
C7—C6—H6	120.6	C11—C12—H12A	109.4
N3—C7—N5	117.3 (2)	N5—C12—H12B	109.4
N3—C7—C6	120.8 (2)	C11—C12—H12B	109.4
N5—C7—C6	121.9 (2)	H12A—C12—H12B	108.0
N5—C8—C9	111.8 (2)	C2—N1—N2	103.6 (2)
N5—C8—H8A	109.3	C3—N2—N1	111.8 (2)
C9—C8—H8A	109.3	C3—N2—C4	128.7 (2)
N5—C8—H8B	109.3	N1—N2—C4	119.4 (2)
C9—C8—H8B	109.3	C7—N3—N4	120.05 (19)
H8A—C8—H8B	107.9	C4—N4—N3	119.34 (19)
C8—C9—C10	111.7 (2)	C7—N5—C12	118.89 (18)
C8—C9—H9A	109.3	C7—N5—C8	120.04 (18)
C10—C9—H9A	109.3	C12—N5—C8	113.3 (2)
			
C3—C1—C2—N1	−0.5 (4)	C5—C4—N2—C3	−169.6 (2)
C2—C1—C3—N2	0.4 (3)	N4—C4—N2—N1	−169.8 (2)
N4—C4—C5—C6	0.4 (4)	C5—C4—N2—N1	11.0 (3)
N2—C4—C5—C6	179.5 (2)	N5—C7—N3—N4	175.5 (2)
C4—C5—C6—C7	−0.7 (4)	C6—C7—N3—N4	−0.9 (3)
C5—C6—C7—N3	1.0 (4)	C5—C4—N4—N3	−0.3 (4)
C5—C6—C7—N5	−175.2 (2)	N2—C4—N4—N3	−179.4 (2)
N5—C8—C9—C10	54.4 (3)	C7—N3—N4—C4	0.5 (3)
C8—C9—C10—C11	−54.9 (3)	N3—C7—N5—C12	1.6 (3)
C9—C10—C11—C12	55.6 (3)	C6—C7—N5—C12	177.9 (2)
C10—C11—C12—N5	−55.7 (3)	N3—C7—N5—C8	148.8 (2)
C1—C2—N1—N2	0.4 (3)	C6—C7—N5—C8	−34.8 (3)
C1—C3—N2—N1	−0.2 (3)	C11—C12—N5—C7	−156.3 (2)
C1—C3—N2—C4	−179.6 (2)	C11—C12—N5—C8	54.3 (3)
C2—N1—N2—C3	−0.1 (3)	C9—C8—N5—C7	157.0 (2)
C2—N1—N2—C4	179.4 (2)	C9—C8—N5—C12	−54.1 (3)
N4—C4—N2—C3	9.6 (4)		
